# Assessment of Nutritional Risk Screening 2002 as predictors of long hospital stay in patients with upper gastrointestinal diseases

**DOI:** 10.3389/fnut.2026.1743320

**Published:** 2026-06-04

**Authors:** Ruiqing Zhu, Xiao Li, Xiuxue Feng, Huikai Li, Fen Deng

**Affiliations:** 1 Department of Gastroenterology, The First Medical Center of Chinese PLA General Hospital, Beijing, China; 2 Department of General Medicine, The Fourth Medical Center of Chinese PLA General Hospital, Beijing, China

**Keywords:** length of hospital stay, malnutrition, nutritional risk, Nutritional Risk Screening 2002, upper gastrointestinal diseases

## Abstract

**Background:**

Among patients admitted to the gastroenterology ward, especially those with upper gastrointestinal (UGI) diseases, malnutrition is markedly prevalent. Nutritional Risk Screening 2002 (NRS2002) is widely used globally to assess the nutritional risk of hospitalized patients.

**Methods:**

A retrospective, single-center study that included 896 patients with UGI diseases was performed in the Department of Esophagogastric‌, Senior Department of Gastroenterology, the First Medical Center of PLA General Hospital. Data from each admitted patient, including demographic information, lifestyle factors, primary diagnosis, comorbidities, laboratory test values at admission and length of hospital stay (LOHS), were extracted from the Electronic Medical Record (EMR) system. An LOHS longer than the 75th percentile was defined as a long LOHS. Univariate and multivariate logistic regression analyses were performed to identify independent risk factors for long LOHS. Subsequently, receiver operating characteristic (ROC) curve analysis was used to assess the discriminative ability of the independent factors calculated for predicting long LOHS.

**Results:**

Among the 896 inpatients in our study, 586 were males and 310 were females, with a median age of 61.0 years. The patients had a median LOHS of 14.0 days (Q1–Q3, 10.3–19.0 days). The inpatients whose LOHS was more than 19 days were classified into the long LOHS group, while others were classified into the non-long LOHS group. On the basis of the univariate and multivariate logistic regression analysis results, both the NRS2002 score and serum albumin level were identified as independent risk factors for long LOHS. An NRS2002 score ≥2 points (optimal cutoff value determined by maximum Youden’s index) reflected good predictive value, with an area under the curve (AUC) of 0.721 according to the ROC analysis.

**Conclusion:**

In conclusion, the results of this study demonstrate that the serum albumin (ALB) concentration and NRS2002 score are predictors of long LOHS in patients with UGI diseases. These findings emphasize the importance of assessing nutritional status in clinical management and may provide guidance for improving the treatment outcomes of patients with UGI diseases.

## Introduction

The upper gastrointestinal tract (UGT) is usually defined as the portion of the digestive tract located above the suspensory ligament of Treitz that extends continuously from the oral cavity through the pharynx, esophagus, and stomach to the proximal segment of the duodenum (1). Upper gastrointestinal (UGI) diseases are traditionally grouped according to their anatomic origin: esophageal, gastric, or duodenal. Commonly encountered conditions include gastroesophageal reflux disease (GERD), Barrett’s esophagus, esophageal adenocarcinoma (EC), gastritis, gastric ulceration, gastric cancer (GC), duodenal ulceration and nonvariceal upper gastrointestinal bleeding (NVUGIB) (1–4). These conditions usually cause difficulties in swallowing, loss of appetite, and sustained catabolic stress, collectively disrupting nutrient intake and utilization and markedly increasing the incidence of malnutrition (5–7). In addition, during the treatment of these diseases, patients may be required to undergo mandatory fasting. For instance, patients with superficial GC who undergo endoscopic submucosal dissection (ESD) must adhere to the prescribed fasting period to ensure proper healing of the mucosa and to prevent iatrogenic bleeding (8).

In China, the average prevalence of malnutrition or nutritional risk among hospitalized patients ranges from 12.5 to 29.4%, depending on the screening tool used and the case combination (9, 10). The prevalence is even higher for cancer patients and elderly patients, reaching 41.3 and 44.1%, respectively (11, 12). Among the elderly patients admitted to the hospital, the proportion of those with nutritional risks in the gastroenterology department is obviously greater than that in any other department (11). Moreover, inadequate dietary intake remains a key contributor to malnutrition, and the Department of Gastroenterology has the highest prevalence of recent and current low food intake (LI_RC_) (13).

Multiple studies have shown that malnutrition is prevalent among hospitalized patients and that it has a negative effect on clinical outcomes, such as poor prognosis, increased infection incidence, prolonged hospital stay and increased mortality rate (9, 14–16). In addition, emerging evidence has demonstrated that providing nutritional support to patients who are at nutritional risk is associated with fewer infectious complications and a shorter length of hospital stay (LOHS), while the resulting incremental cost-effectiveness ratio shows no significant increase in overall treatment costs (17). These findings further highlight the need to conduct nutritional assessments and implement corresponding intervention measures for inpatients in the gastroenterology department.

Nutritional Risk Screening 2002 (NRS2002) is a validated, guideline-endorsed tool that was developed by the European Society for Parenteral and Enteral Nutrition (ESPEN) in 2002 (18). It is currently the most widely adopted nutritional risk screening tool worldwide and was originally designed for hospitalized patients to evaluate the probability that a hospitalized patient will benefit from targeted nutritional intervention (18–20). Although several studies have linked the NRS2002 score to adverse outcomes, such as a prolonged hospital stay and increased in-hospital mortality, its prognostic value, specifically in in-patients with UGI diseases, remains unclear (21–23). Therefore, we designed a retrospective study to explore the relationship between nutritional risk and long LOHS for patients with UGI diseases through the NRS2002.

## Materials and methods

### Study design

**Figure 1:**
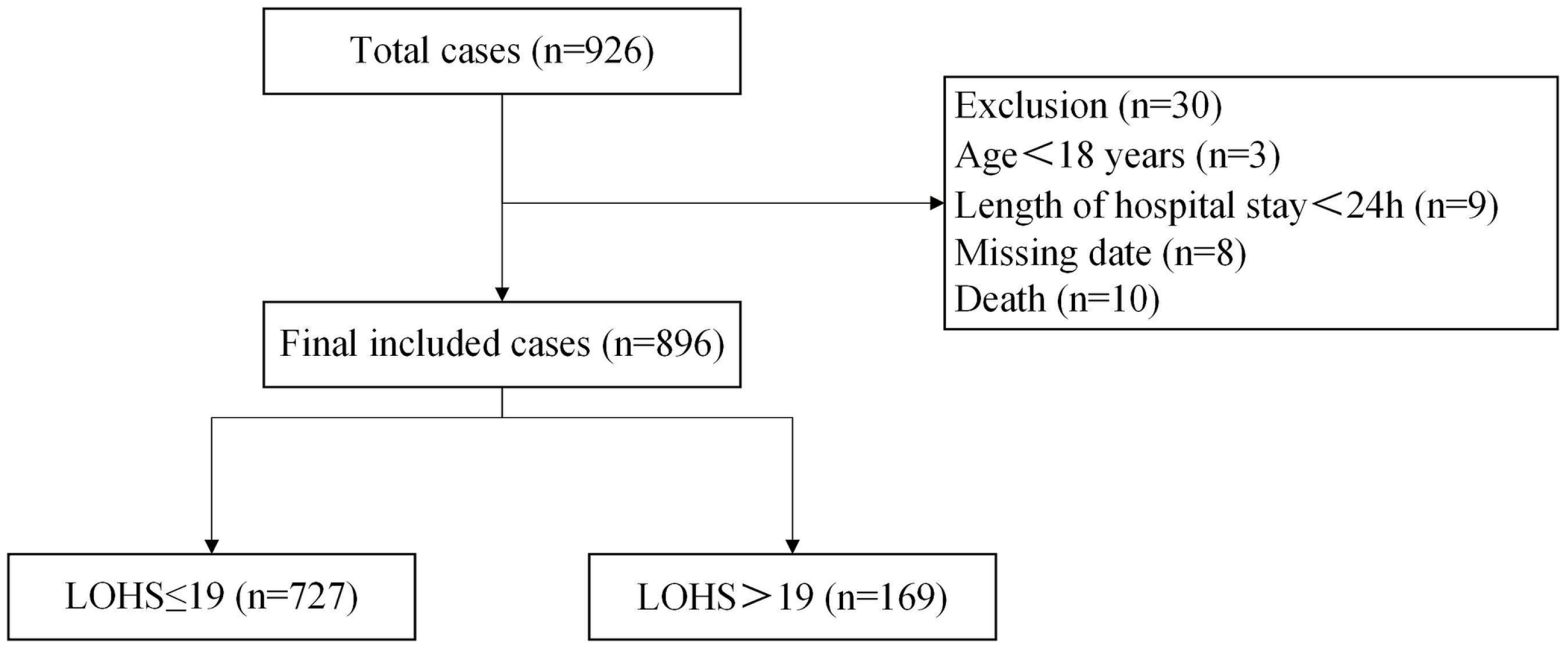
Flow diagram.

A retrospective, single-center cohort study using an electronic medical record (EMR) database was conducted at the First Medical Center of PLA General Hospital. Consecutive hospitalized patients admitted between January 2023 and December 2024 to the Department of Esophagogastric‌, Senior Department of Gastroenterology, of our hospital were screened for eligibility. The inclusion criteria for our study were as follows: (1) a main discharge diagnosis of UGI disease; (2) complete blood cell count and serum chemical tests being completed within 24 h of admission; and (3) NRS2002 assessment being completed within 24 h of admission. The exclusion criteria for our study were as follows: (1) age less than 18 years; (2) length of hospital stay less than 24 h; (3) death during hospitalization; and (4) unavailable clinical data. In accordance with the inclusion and exclusion criteria, among the 926 hospitalized patients initially screened, 30 were excluded; thus, the final analysis sample group consisted of 896 patients (Figure 1). This study was approved by the ethics committee of Chinese PLA General Hospital (Approval No. S2025-351-01).

### Data collection

Data from each admitted patient were extracted from the EMR system, including demographic information [sex, age, and body mass index (BMI)], lifestyle factors (smoking and drinking history), primary diagnosis, comorbidities (hypertension, coronary heart disease (CHD), chronic obstructive pulmonary disease (COPD), diabetes, kidney disease and rheumatoid disease), laboratory test values at admission [red blood cell (RBC) count, hemoglobin (HGB), hematocrit (HCT), white blood cell (WBC) count, neutrophils (NEUT), platelets (PLT), total protein (TP), albumin (ALB), prothrombin time (PT), activated partial thromboplastin time (APTT), and fibrinogen (FIB)] and length of hospital stay. An LOHS longer than the 75th percentile (P75 = 19 days) was defined as a long LOHS.

### Nutritional risk assessment

Within 24 h of admission, the attending physician conducted a systematic assessment of the patient’s nutritional risk using the NRS2002. The NRS2002 systematically evaluates three discrete domains: (1) the severity of the disease, (2) the degree of nutritional impairment, and (3) age. The first two domains are scored using an ordinal scale ranging from 0 to 3, resulting in a cumulative score ranging from 0 to 6, which reflects overall nutritional risk. Patients who reach the age threshold of 70 years or above (≥70 years old) would receive an additional point, and the total score of the final risk assessment ranges from 0 to 7.

### Statistical analysis

Continuous variables are first tested for normality using the Kolmogorov-Smirnov test and visual inspection of Quantile-Quantile Plot (Q–Q plots) (Supplementary Figures S1-S14, Supplementary Table S1). Variables that followed a normal distribution are expressed as the mean ± standard deviation (SD) and were compared with the independent t test. Nonnormally distributed data are expressed as median values (interquartile ranges, Q1–Q3) and were compared using the Mann–Whitney U test. Categorical variables are summarized as frequencies (percentages) and were compared using the *χ*^2^ test or Fisher’s exact test, as appropriate. To quantify the magnitude of between-group differences, effect sizes were planned and calculated according to variable type. For continuous variables, Cohen’s d was considered as a measure of the effect size for mean differences. For categorical variables, Cohen’s h was computed to quantify the effect size of differences in proportions between groups (Supplementary Table S2). Variables with *p* values less than 0.05 in the univariable analysis were entered into a multivariable binary logistic regression model to identify independent factors associated with long LOHS (long LOHS ≥ 75th percentile of the overall cohort). The results are reported as adjusted odds ratios (ORs) with 95% confidence intervals (CIs). A receiver operating characteristic (ROC) curve was constructed, and the area under the curve (AUC) with 95% CI was calculated to evaluate the discriminative ability of the independent factors in predicting long LOHS. The optimal cutoff value was determined by the maximum Youden index (sensitivity + specificity – 1). All the statistical analyses were conducted using IBS SPSS 26.0 and GraphPad Prism 8.0 software, and the statistical results were plotted using GraphPad Prism 8.0. A two-sided *p* value of less than 0.05 was considered to indicate statistical significance.

## Results

### Basic characteristics of 896 patients

A total of 896 patients who were hospitalized from January 2023 to December 2024 in the Department of Esophagogastric‌, Senior Department of Gastroenterology of the First Medical Center of The PLA General Hospital were included in our study. The basic characteristics of these inpatients are presented in Table 1. There were 586 males and 310 females, with a median age of 61.0 years (Q1–Q3, 52.0–70.0 years) and a median BMI of 23.7 kg/m^2^ (Q1–Q3, 20.9–25.7 kg/m^2^). The patients had a median LOHS of 14.0 days (Q1–Q3, 10.3–19.0 days). Therefore, we define a hospitalization period exceeding 19 days as a long LOHS. Inpatients whose LOHS was more than 19 days were classified into the long LOHS group, while others were classified into the non-long LOHS group.

**Table 1:** Baseline characteristics of the included patients.

Variables	Total (*n* = 896)	LOHS ≤ 19 (*n* = 727)	LOHS > 19 (*n* = 169)	*p* value
Sex, male/female (*n*)	586/310	477/250	109/60	0.784
Age, median (Q1–Q3) (year)	61.0 (52.0–70.0)	60.0 (51.0–69.0)	65.0 (56.0–74.0)	0.000
BMI, median (Q1–Q3) (kg/m^2^)	23.7 (20.9–25.7)	23.8 (21.1–25.7)	22.5 (19.0–25.8)	0.003
UGI diseases, [*n* (%)]				
Superficial gastric cancer	227 (25.3)	190 (26.1)	37 (21.9)	0.253
Superficial esophageal cancer	139 (15.5)	112 (15.4)	27 (16.0)	0.854
NVUGIB	103 (11.5)	79 (10.9)	24 (14.2)	0.221
Gastric SMT	90 (10.0)	83 (11.4)	7 (4.1)	0.005
Achalasia	69 (7.7)	60 (8.3)	9 (5.3)	0.198
Advanced gastric or esophageal cancer	56 (6.3)	35 (4.8)	21 (12.4)	0.000
Esophageal SMT	44 (4.9)	41 (5.6)	3 (1.8)	0.036
Duodenal lesion	41 (4.6)	34 (4.7)	7 (4.1)	0.764
UGT stenosis	33 (3.7)	19 (2.6)	14 (8.3)	0.000
UGI variceal bleeding	31 (3.5)	21 (2.9)	10 (6.0)	0.052
GERD	21 (2.3)	18 (2.5)	3 (1.8)	0.795
Others	42 (4.7)	35 (4.8)	7 (4.1)	0.710
Chronic diseases, [*n* (%)]				
Cardiovascular disease	283 (31.6)	216 (29.7)	67 (39.6)	0.012
Respiratory disease	81 (9.0)	48 (6.6)	33 (19.5)	0.000
Diabetes	59 (6.6)	45 (6.2)	14 (8.3)	0.323
Kidney disease	16 (1.8)	7 (1.0)	9 (5.3)	0.000
Rheumatoid diseases	2 (0.2)	2 (0.3)	0 (0.0)	1.000
Smoking history, [*n* (%)]	301 (33.6)	240 (33.0)	61 (36.1)	0.445
Drinking history, [*n* (%)]	302 (33.7)	242 (33.3)	60 (35.5)	0.583
NRS2002, median (Q1–Q3)	1.0 (0.0–2.0)	1.0 (0.0–1.0)	2.0 (1.0–4.0)	0.000

As shown in Table 1, there were 727 patients (81.1%) and 169 patients (18.9%) in the non-long LOHS and LOHS groups, respectively. There was no significant difference between the two groups in terms of sex. However, the patients in the long-LOHS group were relatively older than the patients in the non-long-LOHS group were (*p* = 0.000). The BMI of the patients in the long LOHS group was relatively lower than that of the patients in the non-long LOHS group (*p* = 0.003). Furthermore, the prevalence of UGI diseases, including gastric submucosal tumor (SMT) (*p* = 0.005), advanced gastric or esophageal cancer (*p* = 0.000), esophageal SMT (*p* = 0.036) and upper gastrointestinal tract (UGT) stenosis (*p* = 0.000), differed between the two groups.

In terms of chronic diseases, we found that the proportions of patients with cardiovascular diseases (*p* = 0.012), respiratory system diseases (*p* = 0.000) and kidney diseases (*p* = 0.000) were greater in the long LOHS group than those in the non-long LOHS group. When the smoking history (*p* = 0.445) and drinking history (*p* = 0.583) were analyzed, no statistically significant differences were observed between the two groups. Moreover, a significant difference in the NRS2002 score was detected between the non-long LOHS group and the LOHS group, which was 1.0 (Q1–Q3, 0.0–1.0) and 2.0 (Q1–Q3, 1.0–4.0), respectively (*p* = 0.000).

### Laboratory measurements for patients by length of hospital stay

With respect to laboratory parameters, we found that RBC (*p* = 0.000), HGB (*p* = 0.000), HCT (*p* = 0.000), TP (*p* = 0.000) and ALB (*p* = 0.000) were significantly lower in the long LOHS group than in the non-long LOHS group. In the long LOHS group, the median RBC was 4.0 × 10^9^/L (Q1–Q3, 3.1–4.5 × 10^9^/L), the median HGB was 117.0 g/L (Q1–Q3, 89.5–138.5 g/L), the median HCT was 0.35 (Q1–Q3, 0.26–0.41), the median TP was 63.2 g/L (Q1–Q3, 59.5–67.4 g/L) and the median serum ALB concentration was 37.5 g/L (Q1–Q3, 33.3–41.4 g/L). In the non-long LOHS group, the corresponding values were 4.3 × 10^9^/L (Q1–Q3, 3.9–4.8 × 10^9^/L), 132.0 (Q1–Q3, 118.0–145.0 g/L), 0.39 (Q1–Q3, 0.35–0.42), 65.5 g/L (Q1–Q3, 62.4–69.1 g/L) and 41.2 g/L (Q1–Q3, 38.7–43.4 g/L).

NEUT (*p* = 0.021), PT (*p* = 0.000), APTT (*p* = 0.004) and FIB (*p* = 0.001) were significantly greater in the long LOHS group than in the non-long LOHS group (Table 2). In the long LOHS group, the median NEUT was 3.3 × 10^9^/L (Q1–Q3, 2.2–5.4 × 10^9^/L), the median PT was 13.3 s (Q1–Q3, 12.1–16.1 s), the median APPT was 28.6 s (Q1–Q3, 26.6–35.0 s), and the median FIB was 2.9 g/L (Q1–Q3, 2.4–3.6 g/L). In the non-long LOHS group, the corresponding values were 3.0 × 10^9^/L (Q1–Q3, 2.4–3.9 × 10^9^/L), 12.3 s (Q1–Q3, 11.4–14.3 s), 27.6 s (Q1–Q3, 26.2–31.5 s) and 2.6 g/L (Q1–Q3, 2.3–3.1 g/L).

**Table 2:** Comparison of the laboratory parameters between the two groups.

Variables	Total (*n* = 896)	LOHS ≤ 19 (*n* = 727)	LOHS > 19 (*n* = 169)	*p* value
RBC (×10^9^/L), median (Q1–Q3)	4.3 (3.8–4.8)	4.3 (3.9–4.8)	4.0 (3.1–4.5)	0.000
HGB (g/L), median (Q1–Q3)	130.0 (115.0–144.0)	132.0 (118.0–145.0)	117.0 (89.5–138.5)	0.000
HCT, median (Q1–Q3)	0.38 (0.34–0.42)	0.39 (0.35–0.42)	0.35 (0.26–0.41)	0.000
WBC (×10^9^/L), median (Q1–Q3)	5.5 (4.5–6.7)	5.5 (4.5–6.6)	5.6 (4.1–7.5)	0.687
NEUT (×10^9^/L), median (Q1–Q3)	3.0 (2.4–4.2)	3.0 (2.4–3.9)	3.3 (2.2–5.4)	0.021
PLT (×10^9^/L), median (Q1–Q3)	199.0 (160.0–241.0)	199.0 (161.8–240.0)	203.5 (152.5–243.8)	0.955
TP (g/L), median (Q1–Q3)	65.2 (61.6–68.8)	65.5 (62.4–69.1)	63.2 (59.5–67.4)	0.000
ALB (g/L), median (Q1–Q3)	40.9 (37.9–43.0)	41.2 (38.7–43.4)	37.5 (33.3–41.4)	0.000
PT (s), median (Q1–Q3)	12.5 (11.5–14.8)	12.3 (11.4–14.3)	13.3 (12.1–16.1)	0.000
APTT (s), median (Q1–Q3)	27.9 (26.3–32.2)	27.6 (26.2–31.5)	28.6 (26.6–35.0)	0.004
FIB (g/L), median (Q1–Q3)	2.7 (2.3–3.2)	2.6 (2.3–3.1)	2.9 (2.4–3.6)	0.001

### Multivariate logistic regression analysis of long length of hospital stay

On the basis of the above findings, we conducted univariate logistic regression analysis by selecting relevant variables (Table 3). These variables included age, BMI, gastric SMT, advanced gastric or esophageal cancer, esophageal SMT, UGT stenosis, cardiovascular disease, respiratory disease, kidney disease, RBC, HGB, HCT, NEUT, TP, ALB, PT, APTT, FIB and NRS2002 score. All the significant factors within the univariate logistic regression analysis were included in the multivariable logistic regression analysis (Table 3). Among the various components of the multivariable logistic regression model, only the serum ALB concentration and NRS2002 score were independent factors influencing long LOHS for the inpatients. For every 1-point increase in the NRS2002 score, the probability of long LOHS for inpatients increased by 77.7% (OR = 1.777; CI = 1.485–2.126; *p* = 0.000). Additionally, the serum ALB concentration was negatively correlated with long LOHS for the inpatients (OR = 0.927; CI = 0.867–0.990; *p* = 0.024).

**Table 3:** Analysis of univariate and multivariate logistic regression models to evaluate the relationship of NRS 2002 scores on long LOHS in patients with UGI diseases.

Variables	Univariate (unadjusted)		Multivariate (adjusted)	
	OR (95% CI)	*p* value	OR (95% CI)	*p* value
Age	1.026 (1.013–1.040)	0.000	0.989 (0.973–1.005)	0.173
BMI	0.915 (0.875–0.957)	0.000	1.047 (0.988–1.111)	0.123
Gastric SMT	0.335 (0.152–0.739)	0.007	0.677 (0.294–1.563)	0.361
Advanced gastric or esophageal cancer	2.805 (1.588–4.958)	0.000	0.812 (0.369–1.789)	0.606
Esophageal SMT	0.302 (0.093–0.988)	0.048	0.340 (0.079–1.470)	0.149
UGT stenosis	3.366 (1.652–6.859)	0.001	1.655 (0.692–3.955)	0.257
Cardiovascular disease	1.554 (1.099–2.198)	0.013	1.516 (0.959–2.396)	0.075
Respiratory disease	3.432 (2.124–5.547)	0.000	1.191 (0.641–2.214)	0.580
Kidney disease	5.786 (2.123–15.766)	0.001	1.317 (0.361–4.812)	0.677
RBC	0.583 (0.477–0.712)	0.000	1.789 (0.850–3.762)	0.125
HGB	0.979 (0.973–0.985)	0.000	0.993 (0.971–1.016)	0.570
HCT	0.001 (0.000–0.009)	0.000	0.043 (0.000–6,376.724)	0.605
NEUT	1.130 (1.053–1.213)	0.001	1.052 (0.956–1.156)	0.299
TP	0.955 (0.929–0.981)	0.001	1.006 (0.965–1.049)	0.777
ALB	0.864 (0.833–0.895)	0.000	0.927 (0.867–0.990)	0.024
PT	1.097 (1.038–1.159)	0.001	1.056 (0.984–1.132)	0.129
APTT	1.048 (1.020–1.077)	0.001	1.004 (0.969–1.040)	0.837
FIB	1.433 (1.194–1.720)	0.000	1.232 (0.974–1.559)	0.082
NRS2002	1.875 (1.660–2.118)	0.000	1.777 (1.485–2.126)	0.000

### Receiver operating characteristic curves for albumin and Nutritional Risk Screening 2002

**Figure 2:**
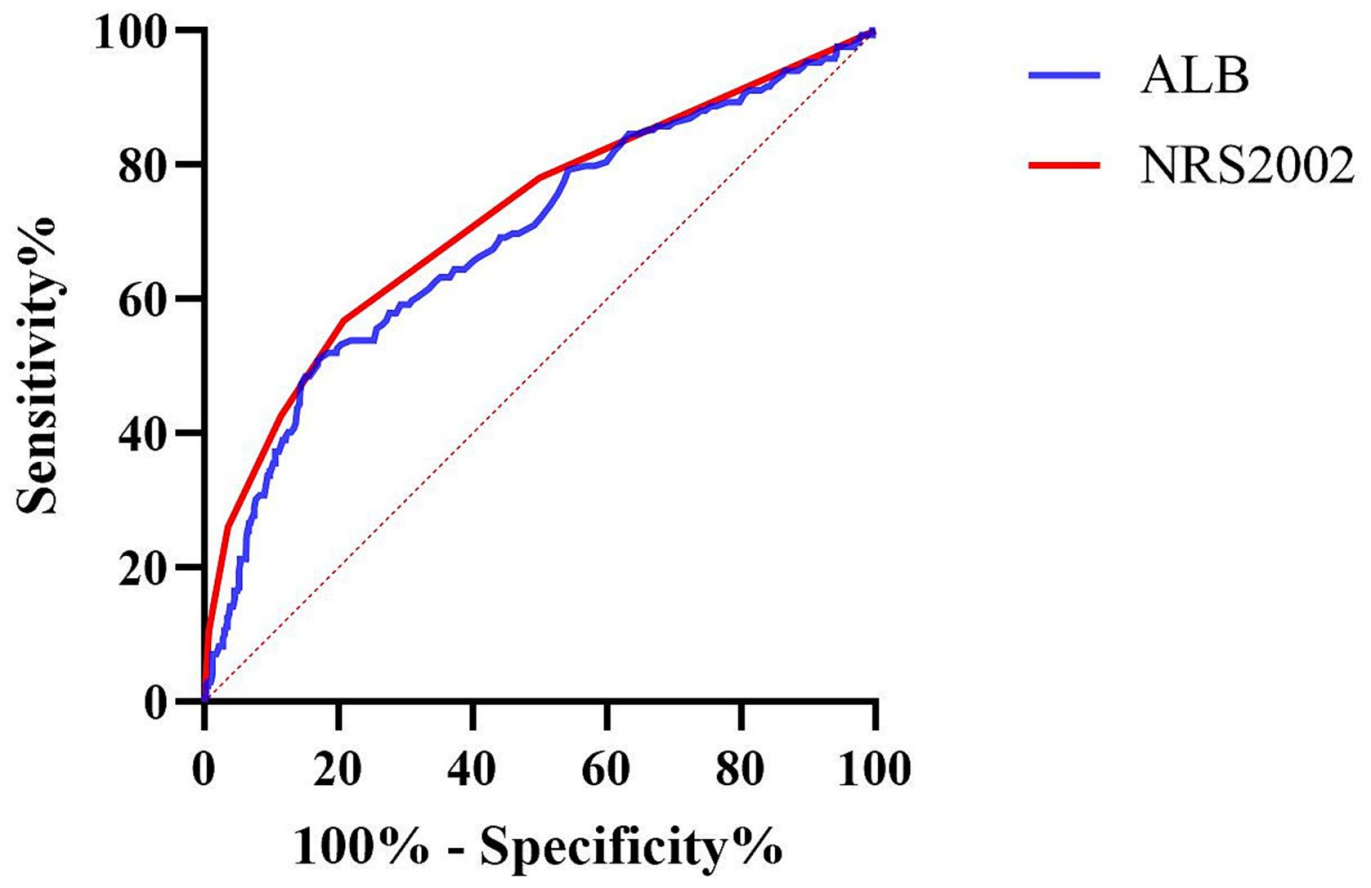
The ROC curves to predict the long LOHS in UGI patients.

The performance of the ALB and NRS2002 scores in predicting long LOHS for inpatients with UGI disease was compared by ROC analysis. As shown in Figure 2, both the serum ALB concentration and the NRS2002 score could reasonably predict long LOHS for inpatients with UGI disease (the ROC curve of the serum ALB concentration and the long LOHS shown in Figure 2 is actually based on the reciprocal of the actual value of the serum ALB concentration). The NRS2002 score was more accurate, with an AUC of 0.721 (95% CI = 0.675–0.768) versus 0.691 (95% CI = 0.644–0.738) for the serum ALB concentration (Figure 2). When the serum ALB concentration was 37.6, the specificity was 0.831, and the sensitivity was 0.509, which were both optimal, as indicated by the highest Youden index. This finding suggests that for inpatients with UGI diseases, using an ALB concentration ≥ 37.6 g/L as a predictor of long LOHS offers an optimal balance of specificity and sensitivity. When the NRS2002 score was 1.5, the specificity was 0.792, and the sensitivity was 0.568, which were both optimal, as indicated by the highest Youden index. This finding suggests that for inpatients with UGI diseases, using an NRS2002 score of ≥ 2 as a predictor of long LOHS offers an optimal balance of specificity and sensitivity.

## Discussion

The LOHS serves as a key barometer of hospital operational efficiency and medical quality (24). Prolonged stays not only occupy hospital beds, increasing the consumption of medical resources (such as medical staff and medical equipment) but also affect the relevant performance evaluation indicators of hospitals and operational efficiency (25). Moreover, such prolonged occupancies can lead to a shortage of medical resources, preventing patients who truly need medical resources from receiving effective treatment (26, 27). A more serious consideration is that a long LOHS may increase the risk of complications and infections for inpatients, and at the same time, lead to a decline in patient satisfaction (27).

Many factors influence inpatient LOHS. The type, size and regional differences of the hospital where the patient is hospitalized may affect the length of hospital stay (28). In addition, patients’ educational level, occupation, and type of medical insurance may influence their medical-seeking behavior and hospitalization decisions, thereby affecting the length of their hospital stay (28, 29). In terms of lifestyle factors, smoking, alcohol consumption and drug use are associated with longer hospital stays for upper gastrointestinal bleeding (UGIB) patients (30). With respect to the nutritional status of inpatients, poor nutritional status is associated with prolonged hospital stays (31, 32).

Addressing malnutrition has become a key priority in global health efforts. NRS2002 is a rapid, low-burden tool for quantifying nutritional risk (33). By conducting routine screenings for all newly admitted patients, medical staff can promptly identify those who are malnourished or at risk of developing such conditions. They can then formulate targeted dietary adjustment plans or provide nutritional supplements for these patients, thereby reducing postoperative complications, shortening LOHS, accelerating the recovery process, and improving patient quality of life and long-term prognosis. A recent study indicated that the incidence of malnutrition risk according to the NRS2002 is approximately 31.2% (34). Multiple studies have shown that early nutritional risk screening for specific nutritional risk groups, such as patients with advanced tumors, elderly patients and critically ill patients, and timely intervention measures can improve prognosis and reduce LOHS (35–38). Inpatients with UGI diseases typically present with a constellation of alarming symptoms, such as anorexia, dysphagia, epigastric pain, bloating, diarrhea, hematemesis, or hematochezia, which may simultaneously suppress appetite, obstruct ingestion, impair intraluminal digestion, and accelerate catabolic loss.

Our study collected data on 896 UGI diseases and analyzed the predictive value of nutritional risk screening for long LOHS in a wide population to test its discriminative power across an undifferentiated, universally admitted population. Furthermore, we defined a long LOHS as hospitalization for patients with UGI diseases lasting longer than the 75th percentile (P75 = 19 days) and compared the data between the two groups: those with a LOHS of more than 19 days and those with a LOHS of 19 days or less. There were significant differences in age, BMI, gastric SMT, advanced gastric or esophageal cancer, esophageal SMT, UGT stenosis, cardiovascular disease, respiratory disease, kidney disease, RBC, HGB, HCT, NEUT, TP, ALB, PT, APTT, FIB and NRS2002 score between the long LOHS group and the non-long LOHS group. After multivariable logistic regression analysis was performed, we found that the serum ALB concentration and NRS2002 score were independent factors influencing long LOHS in patients with UGI diseases. On the basis of the ROC curves of the serum ALB concentration and the NRS2002 score, we found that the NRS2002 had good predictive value for long LOHS, with an AUC of 0.721. That is, an NRS2002 score of UGI diseases in patients ≥ 2 indicates a high risk of long LOHS.

As mentioned above, malnutrition can lead to prolonged LOHS. Multiple studies have shown that the NRS2002 has good predictive value in terms of LOHS and mortality, and the threshold value is set as an NRS2002 score ≥ 3 (22, 23, 39). Given that disease severity and degree of nutritional impairment are the two main components of the NRS2002 assessment, an increase in either of these two indicators is associated with a longer LOS (40). However, the impact of different disease types (such as respiratory diseases or cardiovascular diseases) on LOHS varies significantly. For instance, owing to their high prevalence of comorbidities, severe inflammation, and metabolic disorders, patients with tumors, patients in the intensive care unit (ICU), and elderly patients require a higher NRS2002 threshold to accurately identify high-risk individuals and determine long LOHS among these patients. For patients with UGI diseases, owing to a significant reduction in oral intake and a slow recovery of gastrointestinal function, even if their scores are less than 3 points, the risk of malnutrition significantly increases and significantly affects the LOHS. For hospitalized patients with upper gastrointestinal diseases, an NRS2002 score ≥ 2 may serve as a valuable early warning threshold to identify those at increased risk of prolonged hospitalization. Close nutritional monitoring and further comprehensive nutritional assessment are encouraged to guide decisions regarding early nutritional intervention.

Although this study revealed that the serum ALB concentration and NRS2002 score significantly predict long LOHS for inpatients with UGI diseases, several limitations still exist. First, as our study was retrospective and was conducted at a single center, multicenter prospective studies are necessary to validate our findings. Second, whether nutritional intervention can shorten the LOHS of inpatients with UGI diseases cannot be analyzed in retrospective studies. Third, in our study, the NRS2002 was conducted within 24 h after admission; therefore, continuous and dynamic observational data throughout the entire hospitalization period were lacking. Fourth, the predictive model only exhibits moderate discriminative efficiency based on its AUC value. Additional prospective studies with expanded sample sizes are necessary to further verify the clinical applicability and generalizability of NRS2002 in predicting prolonged LOHS among patients with UGI diseases.

## Conclusion

This study demonstrated that both the serum ALB concentration and the NRS2002 score are potential predictors of long LOHS for patients admitted with UGI diseases.

## Data Availability

The original contributions presented in the study are included in the article/Supplementary material, further inquiries can be directed to the corresponding authors.
