# Supplementary material for: Assessment of Nutritional Risk Screening 2002 as predictors of long hospital stay in patients with upper gastrointestinal diseases
Source: Front Nutr. 2026 Jun 4;13:1743320. doi: 10.3389/fnut.2026.1743320 (PMC13275261; doi:10.3389/fnut.2026.1743320)
Supplement: Supplementary file 15 [file Supplementary_File_1.docx]

Supplementary Figure S1 The normal and detrended Q-Q plots of Age.

A The normal Q-Q plot of age. B The detrended normal Q-Q plot of age.

Supplementary Figure S2 The normal and detrended Q-Q plots of BMI.

A The normal Q-Q plot of BMI. B The detrended normal Q-Q plot of BMI.

Supplementary Figure S3 The normal and detrended Q-Q plots of NRS2002.

A The normal Q-Q plot of NRS2002. B The detrended normal Q-Q plot of NRS2002.

Supplementary Figure S4 The normal and detrended Q-Q plots of RBC.

A The normal Q-Q plot of RBC. B The detrended normal Q-Q plot of RBC.

Supplementary Figure S5 The normal and detrended Q-Q plots of HGB.

A The normal Q-Q plot of HGB. B The detrended normal Q-Q plot of HGB.

Supplementary Figure S6 The normal and detrended Q-Q plots of HCT.

A The normal Q-Q plot of HCT. B The detrended normal Q-Q plot of HCT.

Supplementary Figure S7 The normal and detrended Q-Q plots of WBC.

A The normal Q-Q plot of WBC. B The detrended normal Q-Q plot of WBC.

Supplementary Figure S8 The normal and detrended Q-Q plots of NEUT.

A The normal Q-Q plot of NEUT. B The detrended normal Q-Q plot of NEUT.

Supplementary Figure S9 The normal and detrended Q-Q plots of PLT.

A The normal Q-Q plot of PLT. B The detrended normal Q-Q plot of PLT.

Supplementary Figure S10 The normal and detrended Q-Q plots of TP.

A The normal Q-Q plot of TP. B The detrended normal Q-Q plot of TP.

Supplementary Figure S11 The normal and detrended Q-Q plots of ALB.

A The normal Q-Q plot of ALB. B The detrended normal Q-Q plot of ALB.

Supplementary Figure S12 The normal and detrended Q-Q plots of PT.

A The normal Q-Q plot of PT. B The detrended normal Q-Q plot of PT.

Supplementary Figure S13 The normal and detrended Q-Q plots of APTT.

A The normal Q-Q plot of APTT. B The detrended normal Q-Q plot of APTT.

Supplementary Figure S14 The normal and detrended Q-Q plots of FIB.

A The normal Q-Q plot of FIB. B The detrended normal Q-Q plot of FIB.
