# Supplementary material for: Assessment of Nutritional Risk Screening 2002 as predictors of long hospital stay in patients with upper gastrointestinal diseases
Source: Front Nutr. 2026 Jun 4;13:1743320. doi: 10.3389/fnut.2026.1743320 (PMC13275261; doi:10.3389/fnut.2026.1743320)
Supplement: Supplementary file 16 [file Table_1.docx]

Supplementary Table S1 Normality test results for continuous variables

| Variables | Kolmogorov‑Smirnov test | Shapiro–Wilk test |
| --- | --- | --- |
| Age (year) | 0.000 | 0.000 |
| BMI (kg/m^2^) | 0.000 | 0.000 |
| NRS2002 | 0.000 | 0.000 |
| RBC (×10^9^/L) | 0.000 | 0.000 |
| HGB (g/L) | 0.000 | 0.000 |
| HCT | 0.000 | 0.000 |
| WBC (×10^9^/L) | 0.000 | 0.000 |
| NEUT(×10^9^/L) | 0.000 | 0.000 |
| PLT (×10^9^/L) | 0.000 | 0.000 |
| TP (g/L) | 0.000 | 0.000 |
| ALB (g/L) | 0.000 | 0.000 |
| PT (s) | 0.000 | 0.000 |
| APTT (s) | 0.000 | 0.000 |
| FIB (g/L) | 0.000 | 0.000 |
