# Supplementary material for: Assessment of Nutritional Risk Screening 2002 as predictors of long hospital stay in patients with upper gastrointestinal diseases
Source: Front Nutr. 2026 Jun 4;13:1743320. doi: 10.3389/fnut.2026.1743320 (PMC13275261; doi:10.3389/fnut.2026.1743320)
Supplement: Supplementary file 17 [file Table_2.docx]

Supplementary Table S2 Cohen's h and 95% confidence intervals for categorical variables

| Variables | *p* value | Cohen's h | 95% CI |
| --- | --- | --- | --- |
| Sex | 0.784 | 0.0229 | −0.1445-0.1903 |
| UGI diseases |  |  |  |
| Superficial gastric cancer | 0.253 | 0.0996 | −0.0678–0.2670 |
| Superficial esophageal cancer | 0.854 | −0.0154 | −0.1828-0.1520 |
| NVUGIB | 0.221 | −0.1004 | −0.2678-0.0670 |
| Gastric SMT | 0.005 | 0.2794 | 0.1120-0.4468 |
| Achalasia | 0.198 | 0.1162 | −0.0512-0.2836 |
| Advanced gastric or esophageal cancer | 0.000 | −0.2776 | −0.4450-−0.1102 |
| Esophageal SMT | 0.036 | 0.2124 | 0.0450-0.3798 |
| Duodenal lesion | 0.764 | 0.0266 | −0.1408-0.1940 |
| UGT stenosis | 0.000 | −0.2596 | −0.4270-−0.0922 |
| UGI variceal bleeding | 0.052 | −0.1498 | −0.3172-0.0176 |
| GERD | 0.795 | 0.0490 | −0.1184-0.2164 |
| Others | 0.710 | 0.0312 | 0.0726-0.4074 |
| Chronic diseases |  |  |  |
| Cardiovascular disease | 0.012 | −0.2152 | 0.0478-0.3826 |
| Respiratory disease | 0.000 | -0.4026 | 0.2352-0.5700 |
| Diabetes | 0.323 | −0.0794 | −0.0880-0.2468 |
| Kidney disease | 0.000 | -0.2664 | −0.4338-−0.0990 |
| Rheumatoid diseases | 1.000 | 0.1054 | −0.0620-0.2728 |
| Smoking history | 0.445 | −0.0638 | −0.1036-0.2312 |
| Drinking history | 0.583 | −0.0468 | −0.1206-0.2142 |
